# Usefulness of endovascular stent-graft for combination with a strict aort coarctation and patent ductus arteriosus for an adult patient: a case report

**DOI:** 10.4076/1757-1626-2-8477

**Published:** 2009-09-15

**Authors:** Mustafa Kemal Batur, Mevlut Koç, Ilkut Ozer, Gulcan Abalı

**Affiliations:** 1 Department of Cardiology, Adana Numune Education and Research Hospital, Adana, 01330, Turkey

## Abstract

We reported a 21-year-old patient with strict descending aorta coarctation and small post-ductal patent ductus arteriosus, complicated with abortion and hypertension. The patient was successfully treated by endovascular stent-graft with a single cardiac catheterization. Endovascular stent-graft is an easy, safe, and reliable intervention for the treatment of strict descending aorta coarctation and small post-ductal patent ductus arteriosus.

## Introduction

Coarctation of the thoracic aorta is a common congenital heart disease (approximately 6-8%) and it is associated with other congenital cardiac defects, particularly bicuspid aortic valve, mitral valve abnormality, and ventricular septal defect [1]. Rarely coarctation of the thoracic aorta may occur in combination with patent ductus arteriosus (PDA) [2]-[5].

Endovascular stenting is rapidly becoming preferred intervention for the thoracic aorta disease, such as coarctation, aneurysm, and dissection. A recent study was shown that, primer angioplasty or stenting and surgical repair have similar efficacy for the treatment of aortic coarctation [6]. Combination of aortic coarctation and PDA can be treated with therapeutic catheterization and several types of devices [3]-[5].

Herein, we reported a case with descending aortic coarctation and PDA combination, which is successfully treated by using endovascular stent-graft on a single angiographic catheterization.

## Case presentation

A 21-year-old white Turkish woman referred to our department for the investigation of new onset hypertension developed one week after a miscarriage. Past medical history was insignificant except for miscarriage and there was no history of hypertension before and during pregnancy period. Physical examination revealed weak femoral pulses and brachiofemoral delay. Right and left arm, and leg blood pressures were 190/110 mmHg, 140/90 mmHg, and 80/50 mmHg, respectively. A grade 3/6 systolic murmur was heard over the precordial and interscapular areas.

**Figure 1:**
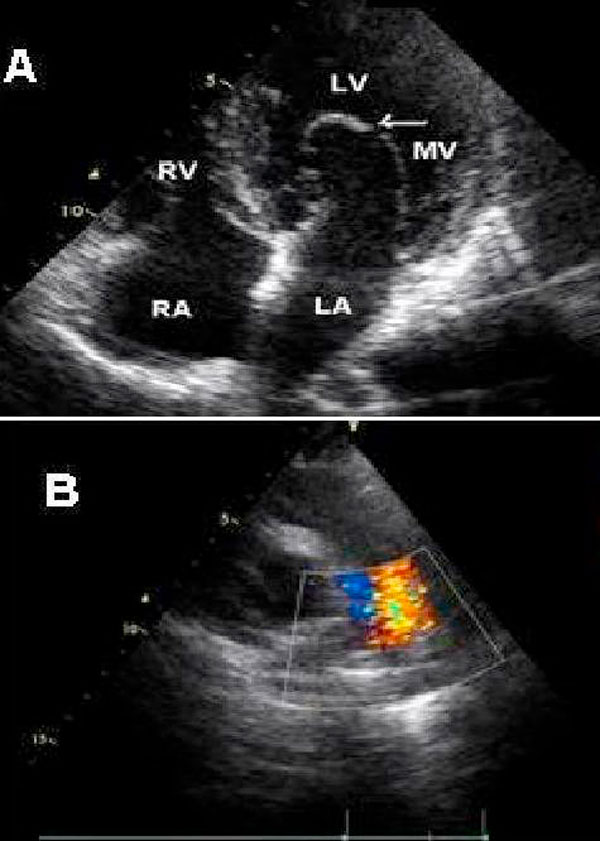
**Abnormal mitral valve with congenital elongation and patent ductus arteriosus was shown apical four chamber view (A) and parasternal short axe view (B), respectively**.

**Figure 2:**
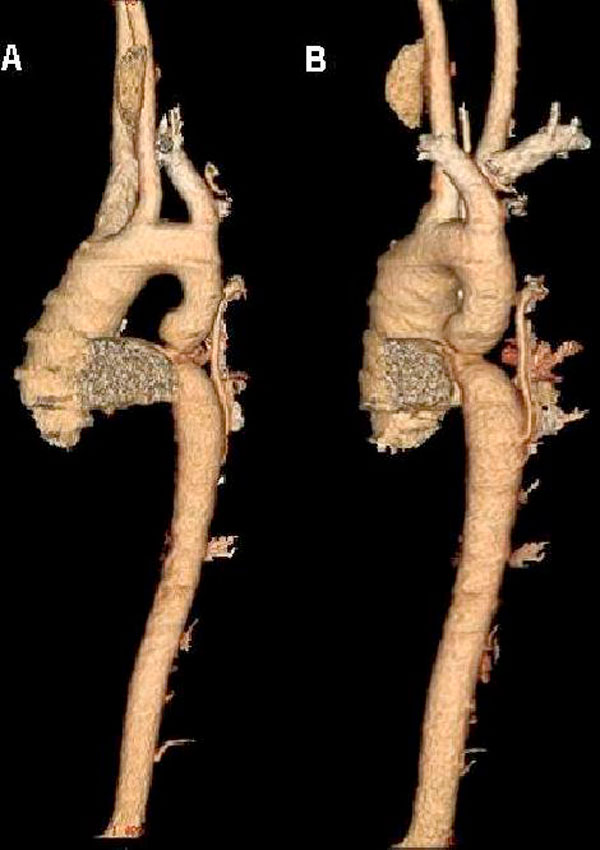
**Thorax CT angiography in anterior posterior (A) and left anterior oblique projections (B), a strict aortic coarctation proximal part of descending aort, ascending aort dilatation, a small size patent ductus arteriosus, and widening and tortuous intercostals artery was detected**.

The electrocardiogram was normal. Rib notching was evident on chest radiography. Standard and Doppler echocardiographic findings were normally functioning bicuspid aortic valve, abnormal mitral valve with congenital elongation, coarctation of descending aorta with a measured maximum gradient of 93 mmHg across the coarctation and mild sized PDA (Figure 1). Thorax CT angiography showed a strict aortic coarctation in proximal part of descending aorta, a small sized PDA, and widening and tortuosity in intercostal arteries (Figure 2). The measured coarctation length was 12 mm, isthmus was 19 mm, smallest diameter of aortic coarctation was 5 mm, and postcoarctation aortic diameter was 21 mm.

**Figure 3:**
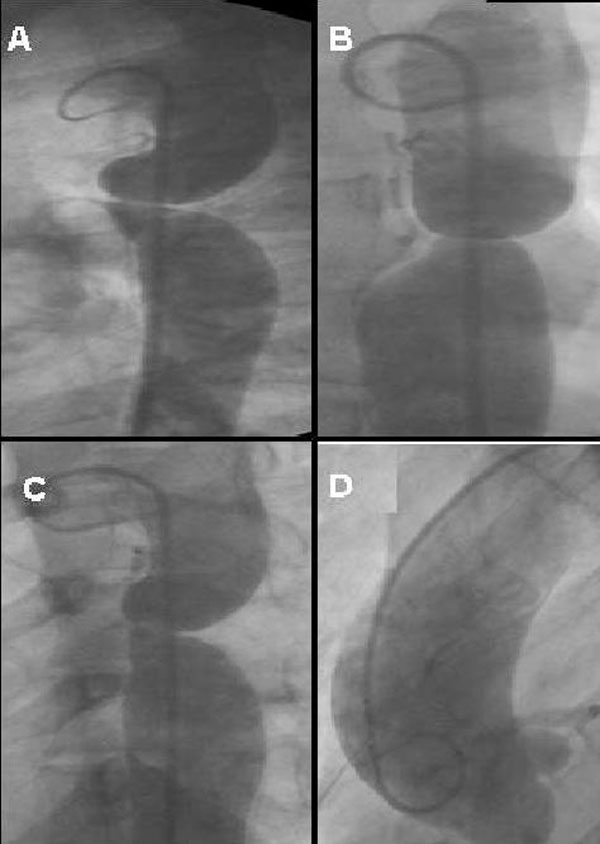
**Descending aortography in the lateral projection (A), a patent ductus arteriosus and strict aortic coarctation was showed better than other projection, in the right oblique projection (B) shows widening and tortuous intercostals artery, in anterior posterior projection (C) strict aortic coarctation ascending aort and widening and tortuous intercostals artery was showed, and ascending aortogram in anterior posterior projection (D) shows minimal dilatation ascending aort**.

Cardiac catheterization was performed under general anesthesia and right femoral artery and left femoral vein cannulated. The coarctation segment could not be crossed with 0.035 diagnostic guide wire. Therefore, we used the 0.014 hydrophilic guide wire for crossing the coarctation segment. Diagnostic multiple pores and pigtail catheter were passed across the coarctation over the hydrophilic guide wire. Measurements of aorta and coarctation segment were similar with the CT angiography. The maximal gradient across the coarctation was 102 mmHg and Qp/Qs: 1.2/1. Arcus aorta, ascending aorta and aortic branches were normal in anatomy but ascending aorta was minimally dilated (39 mm; Figure 3).

**Figure 4:**
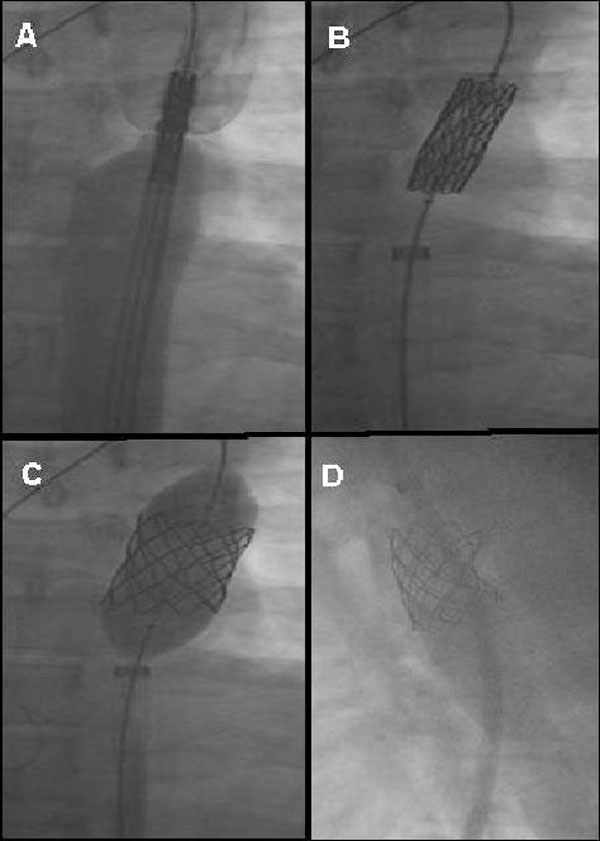
**Deployment of stent, firstly (A) the stent-graft was checking for correct position, secondly (B) the inner balloon was inflated, thirdly (C) outer balloon was inflated fully**. Control aortography showed that **(D),** stent-graft in position covering the coarctation segment and complete occlusion of the patent ductus arteriosus was achieved.

**Figure 5:**
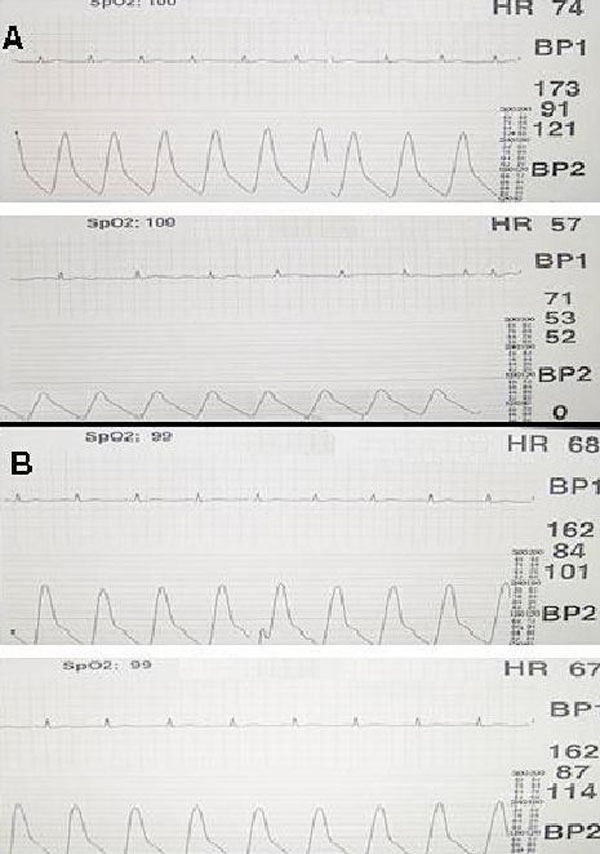
**Before endovascular graft stent, maximum gradient across the coarctation was 102 mmHg (A), The gradient across the coarctation was decreased and measured as 0 mmHg after the procedure (B)**.

Pigtail catheter was changed to a 14F, 75 cm long Mullins sheath over the 0.035 exchange guide wire. A custom-made eight-zig, 45 mm long CP covered stent (NuMed, Hopkinton, NY) loaded on a BIB balloon (inner balloon 12 mm × 9 mm, outer balloon 22 mm × 4 cm) was used. After attachment, the excess covering material is folded around the stent. The graft stent and balloon assembly was passed through the sheath, after checking for correct positioning. Firstly, the inner balloon was inflated and the position was rechecked by angiography and then the outer balloon was inflated fully (Figure 4). Both balloons were then deflated, with the inner one being deflated first before being withdrawn through the sheath. Control aortography showed that stent-graft in position covering the coarctation segment and complete occlusion of the patent ductus arteriosus was achieved without any complication. Maximum gradient across the coarctation was measured as 30 mmHg. Therefore, stent-graft was over dilated using high-pressure balloon (22 mm × 4 cm, NuMed, Hopkinton, NY). The gradient across the coarctation was decreased and measured as 0 mmHg (Figure 5). The coarctation segment of aorta was increased from 6 mm to 19 mm. The procedure was completed without complication.

After the procedure, patient was monitored for 48 hours for possible complications and blood pressure control. Blood pressure was returned to normal limits as were the discrepancy between arm and legs. No anti-hypertensive medication was needed after the procedure and the patient was discharged with 300 mg acetyl salicylate. The patient was followed for eight months without any problem and on her last visit repeat catheterization showed no gradient across the grafted segment or evidence of PDA.

## Discussion

Coarctation of the thoracic aorta is a common congenital heart defect, and consists of the localized shelf in the posteriolateral aortic wall opposite to the ductus arteriosus [1]. Complex coarctation is defined as coarctation in the presence of other important intracardiac abnormalities (e.g., ventricular septal defect, mitral stenosis and bicuspid aorta with stenosis). Simple coarctation refers to coarctation in the absence of such lesions and most common form detected de novo in adults [1]. Complex coarctation and combination with patent ductus arteriosus was uncommon in adults and presented only in case reports [3,4,7]. Significant coarctation requires a gradient greater than 20 mmHg across the coarctation segment of angiography with or without hypertension. Our patient had a complex coarctation with post-ductal PDA and had a peak gradient of 102 mmHg. Unless treated coarctation has a poor prognosis and most patients die from stroke, coronary artery disease or sudden death by the fourth decade [8].

Aorta coarctation is an unusual cause of hypertension in pregnancy however 58% of patients with significant coarctation develop hypertension during pregnancy [9]. Distally decreased blood pressure due to aortic stenosis causes placental hypoperfusion, therefore recurrent abortion is not uncommon in this population [10,11].

A recent study suggested that primary angioplasty or stenting and surgical repair has similar efficacy in treating patients with native aorta coarctation [12]. One approach is closure of PDA with coils or amplatzer duct occluder and correction of coarctation by balloon dilatation or stenting either simultaneously or sequentially [4,13]. This technique can be used in pediatric population however in adults' serious complications like aneurysm, dissection, hematoma, and rupture of aorta may ensue [13].

More recently, percutaneous intraluminal stent-graft placement by transluminal catheter technique was introduced as an alternative intervention in two independent case reports [3,5]. The aim of procedure is to close entry site of PDA. Both cases were successfully treated without any complication. In these reports, both PDAs were pre-ductal, one was being moderate the other one was large sized. Our case had a small-sized, post-ductal PDA and complete occlusion was achieved after percutaneous intraluminal stent-graft without any complication.

## Conclusion

Transluminal endovascular stent-graft placement is an effective, easy to perform method for the treatment of aortic coarctation combined with post-ductal PDA even for adult patients. This technique has advantages of being far less invasive than surgery and reduced exposure time to fluoroscopy, therefore we believe that it would be widely preferred technique in the future for the correction of such anomalies.

## Consent

Written informed consent was obtained from the patient for publication of this case report and accompanying images. A copy of the written consent is available for review by the Editor-in-Chief of this journal.

## Competing interest

The authors declare that they have no competing interests.

## Authors' contributions

IO was involved in the clinical care of the patient. MK researched, wrote the paper and revised the final manuscript. MKB and GA supervised the manuscript and treated the patient. All authors read and approved the final manuscript.

## References

[B1] Webb GD Smallhorn JF Therrien J Redington AN Peter LRobert OBDouglas LMDouglas PZEugene BPhiladelphia WB Textbook of Cardiovasculer Medicine 2007 8 Saunders Elsevier 1605 1608

[B2] Fyler DC Buckley LP Hellenbrand WE Cohen HE Report of the new England regional infant cardiac program Paediatrics 1980 65 376 461

[B3] Sadiq M Malick NH Qureshi SA Simultaneous treatment of native coarctation of the aorta combined with patent ductus arteriosus using a covered stent Catheter Cardiovasc Interv 2003 59 387 390 10.1002/ccd.10501 12822166

[B4] Celebi A Yalçin Y Erdem A Zeybek C Akdeniz C Polat TB Successful transcatheter balloon dilatation of coarctation of aorta and coil occlusion of patent ductus arteriosus in a single catheterization procedure Pediatrics 1980 65 375 461 17479653

[B5] Kulkarni S Vimala J Parmar R Single therapeutic catheterization for treatment of native coarctation of aorta and large patent ductus arteriosus using a covered stent Indian Heart J 2005 57 713 716 16521644

[B6] Mahadevan V Mullen MJ Endovascular management of aortic coarctation Int J Cardiol 2004 97 75 78 10.1016/j.ijcard.2004.08.011 15590082

[B7] Ing FF McMahon WS Johnson GL Vick GW Mullins CE Single therapeutic catheterization to treat coexisting coarctation of the aorta and patent ductus arteriosus Am J Cardiol 1997 79 535 537 10.1016/S0002-9149(96)00807-7 9052372

[B8] Maron BJ Humphries JO Rowe RD Mellits ED Prognosis of surgically corrected coarctation of the aorta. A 20-year postoperative appraisal Circulation 1973 47 119 126 4686589 10.1161/01.cir.47.1.119

[B9] Beauchesne LM Connolly HM Ammash NM Warnes CA Coarctation of the aorta: outcome of pregnancy J Am Coll Cardiol 2001 38 1728 1733 10.1016/S0735-1097(01)01617-5 11704388

[B10] Mortensen JD Ellsworth HS Coarctation of the aorta and pregnancy: obstetric and cardiovascular complications before and after surgical correction JAMA 1965 191 596 598 14239034 10.1001/jama.1965.03080070080017

[B11] Inmon TW Pollock BE Coarctation of abdominal aorta: review of the literature Am Heart J 1956 53 314 10.1016/0002-8703(56)90269-1 13339698

[B12] Carr JA The results of catheter-based therapy compared with surgical repair of adult aortic coarctation J Am Coll Cardiol 2006 47 1101 1107 10.1016/j.jacc.2005.10.063 16545637

[B13] Liang CD Ko SF Tiao MM False aneurysm and mediastinal hematoma: complications of simultaneous transcatheter therapy for coarctation of the aorta and patent ductus arteriosus in an infant J Invasive Cardiol 2001 13 710 712 11581516

